# Changes in Community Mobility in Older Men and Women. A 13-Year Prospective Study

**DOI:** 10.1371/journal.pone.0087827

**Published:** 2014-02-07

**Authors:** Sofi Fristedt, Anna K. Dahl, Anders Wretstrand, Anita Björklund, Torbjörn Falkmer

**Affiliations:** 1 School of Health Sciences, Jönköping University, Jönköping, Sweden; 2 Department of Medical Epidemiology and Biostatistics, Karolinska Institutet, Stockholm, Sweden; 3 Institute of Gerontology, School of Health Sciences, Jönköping University, Jönköping, Sweden; 4 Faculty of Engineering, Lund University, Lund, Sweden; 5 School of Occupational Therapy and Social Work, Curtin University, Perth, Australia; 6 Rehabilitation Medicine, Department of Medicine and Health Sciences (IMH), Faculty of Health Sciences, Linköping University and Pain and Rehabilitation Centre, UHL, County Council, Linköping, Sweden; 7 School of Occupational Therapy, La Trobe University, Melbourne, Australia; University of Rome Foro Italico, Italy

## Abstract

Community mobility, defined as *“moving [ones] self in the community and using public or private transportation”,* has a unique ability to promote older peoples’ wellbeing by enabling independence and access to activity arenas for interaction with others. Early predictors of decreased community mobility among older men and women are useful in developing health promoting strategies. However, long-term prediction is rare, especially when it comes to including both public and private transportation. The present study describes factors associated with community mobility and decreased community mobility over time among older men and women. In total, 119 men and 147 women responded to a questionnaire in 1994 and 2007. Respondents were between 82 and 96 years old at follow-up. After 13 years, 40% of men and 43% of women had decreased community mobility, but 47% of men and 45% of women still experienced some independent community mobility. Cross-sectional independent community mobility among men was associated with higher ratings of subjective health, reporting no depression and more involvement in sport activities. Among women, cross-sectional independent community mobility was associated with better subjective health and doing more instrumental activities of daily living outside the home. Lower subjective health predicted decreased community mobility for both men and women, whereas self-reported health conditions did not. Consequently, general policies and individual interventions aiming to improve community mobility should consider older persons’ subjective health.

## Introduction

Defined as *“…moving self in the community and using public or private transportation…”*, [1] community mobility (CM) promotes older peoples’ wellbeing and autonomy by enabling independence [2]. The present study focuses on the ability to transport oneself beyond walking distance, including use of private or public transportation (PT), and ability to walk to and from a vehicle at a destination as means of CM. Community mobility has considerable importance by facilitating access to activity arenas to enable interactions with others [2], [3]. Restricted CM may thus reduce social contacts, negatively affect mental health and wellbeing and lead to social exclusion [4]. Consequently, maintaining CM is a positive health goal of vital importance [4], [5]. Early identification of CM decline may guide interventions and general policies aiming to promote older peoples’ health. However, such early identification is contingent upon knowledge about factors influencing CM in older men and women from a long-term perspective. Unfortunately, little is known about potentially important factors for sustained CM over time in a sample using both private transportation and PT.

Existing studies have primarily focused on mobility limitations in relation to walking, consequently only defining mobility limitations and disregarding various modes of transportation. However, mobility limitations may indicate important factors to investigate in relation to CM. For example, previous research has demonstrated that lower financial resources, low social participation [6] and “tiredness in daily activities” [7] increase the risk of limited mobility in both men and women. Moreover, low social participation, poor psychological functioning and low physical activity predict mobility limitations in men, whereas home help and low physical activity predict mobility limitations in women [7]. Lower education levels, especially in women, are known to be associated with increased risk of experiencing disability and functional limitations in later life [8]. Gender differences in old age have also been related to declining health and disability, including mobility [9]–[11]. Mobility becomes more complicated when an individual must travel beyond walking distance and consequently multiple factors (e.g., financial, psychosocial, environmental, physical and cognitive) may interact and impact CM [12].

A recent study of private transport identified risk factors for mobility limitations and driving cessation, namely; older age, female gender, cognitive impairment, low physical activity, reduced balance and impaired gait [13]. In addition, chronic conditions and low functional self-efficacy were risk factors for mobility limitations, and severe visual impairment, weight loss, and slower gross motor coordination were risk factors for driving cessation.

There are also gender-specific differences in the relationships between private transportation, PT and CM. Older women are especially vulnerable for CM limitations, as a result of their needs not being met by PT after driving cessation [14]–[16]. Other studies have focused on health-related factors associated with mobility limitations in older men and women. However, these studies focused on short time periods and few have taken a long-term perspective in relation to influences on CM. Knowledge of long-term predictors of sustained CM in later-life could be extremely valuable to inform health-sustaining practices. Hence, the aim of the present study was to describe factors associated with CM, as well as decreased CM over time, among older men and women. The study also aimed to investigate possible gender differences specific to CM in older adults.

## Methods

### Ethics Statement

Approval for data collection was obtained from the Swedish Data Inspection Office and the Ethical Committee of the Karolinska Institute in Stockholm, Sweden.

### Participants and Study Design

**Figure 1:**
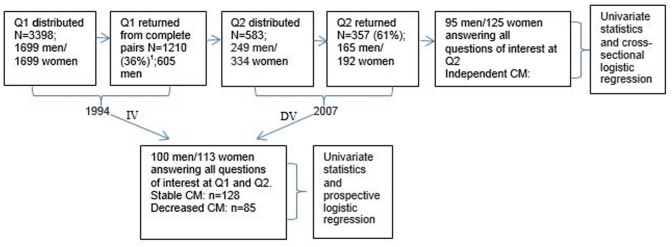
Data and analyses in the present study. (IV = Independent variables, DV = dependent variable, CM = Community mobility) ^1^ Only twin pairs in which both twins had returned their questionnaires were included at Q1; in total 1843 were returned (54% response rate).

Gender-balanced data from a project entitled “Aging in men and women: a longitudinal study of gender differences in health behavior and health among elderly” (GENDER) [9] based on pairs of unlike-sex twins were utilized. Full selection criteria and characteristics of the sample are described elsewhere [9]. In short, the sample consisted of unlike-sex twins born between 1906 and 1925, and whose birth records had been collected between 1959 and 1961, in order to establish a twin registry for epidemiological research. The baseline data of GENDER were collected by Questionnaire 1 (Q1) in 1994 and the follow-up data by Questionnaire 2 (Q2) in 2007 (Figure 1).

### Variables

#### Community Mobility (CM)

CM was assessed at Q1 and Q2, from the same question *“Are you able to transport yourself to places beyond walking distance”,* i.e., CM by private or public transport, and including walking to and from the vehicle at origin and destination. The three original response steps [yes/yes, with some problems/no] were subsequently dichotomised in line with previous research [6], [17] and became; independent CM [yes] and restricted CM [yes, with some problems/no]. As only three men and 14 women in Q1 experienced restricted CM, no cross-sectional analyses were performed on Q1.

Change in CM was created from the dichotomised CM variables at Q1 and Q2. All changes in CM were negative. Among those who responded to Q2, four participants had restricted CM at both Q1 and Q2 and were removed from further analyses. In Q2, additional questions regarding current main mode of transport for CM and main mode of transport two years prior (i.e., in 2005) were available. These questions were, however, not included in first questionnaire (Q1).

#### Independent variables

The main independent variables were informed by previous research mentioned earlier [9], [11], [17]. Namely;

- chronological age;
- marital status; being married ( = 1) or not married ( = 2), including divorced and widowed;
- educational level; ranging from less than elementary school to ( = 1) university degree ( = 6);
- subjective economy; based on to what degree the participants felt that their economic situation prevented them from doing what they wanted: no ( = 1), yes, somewhat ( = 2), yes, a lot ( = 3);
- subjective health based on the survey question “How do you consider your general health condition to be?”; measured by a three-point scale: good ( = 1), fairly good ( = 2), bad ( = 3);
- social network; based on to what degree the participants felt that they were part of a social network: yes, very much ( = 1), yes, somewhat ( = 2), no, hardly ( = 3), no, not at all ( = 4);
- sport activities; frequency of taking part in sport activities such as jogging, playing golf, tennis, etc.: at least once a week ( = 1), less than once a week ( = 2), and;
- I-ADL (Instrumental Activities of Daily Living) outside home; frequency of shopping, going to the bank, pharmacy, etc.: daily ( = 1), once a week ( = 2), once a month ( = 3), less than once a month ( = 4), never ( = 5).

### Index Variables

An index of community activities was constructed based on three questions with respect to self-reported frequency of taking part in social club meetings, church activities or extra curriculum courses. The response options ranged from daily ( = 1), once a week ( = 2), once a month ( = 3), less than once a month ( = 4) to never ( = 5).

Three indices for health conditions were constructed. Firstly, a self-reported index of cerebrovascular conditions was created by respondents indicating the presence or absence of the following; stroke, heart failure, myocardial infarction and angina pectoris. Positive indications were scored as one with a maximum total for this index being four. Secondly, a self-reported index of musculoskeletal conditions including rheumatoid arthritis, knee problems, sciatic problems, hip problems, osteoporosis and gout was created with a maximum possible total of six. Self-reported eye conditions were also indexed and included cataract, glaucoma and other eye conditions in Q1, and cataract, glaucoma and age related macular degeneration in Q2. This index had a maximum possible total of three at each time period.

A single index for all these health conditions considered most likely to influence CM was tested, however, it did not improve the models’ fit and was discarded.

Finally, an index based on the CES-D (Centre for the Epidemiologic Studies Depression Scale) scale was created [18]. The CES-D scale assesses self-reported frequencies of depressive symptoms during the past month (never or almost never/rather seldom/rather often/always or almost always) and the 20 questions are summed to create a total score. This total score was then used to create a dichotomous depression index to ensure similar levels of variance between this variable and its covariates [19]. CES-D total scores were split at 16. Totals of 16 and above were recoded as 2 and indicated high prevalence of depressive symptoms [18]. Scores below 16 were recoded as 1 and indicated few or non-existent depressive symptoms [18].

### Statistical Analyses

SPSS version 19.0 was used to perform all statistical analyses. χ^2^ tests were used to analyse gender differences with dichotomous variables and Mann-Whitney U-tests were used for the remaining variables. Wilcoxon Signed Rank Tests were used to analyse change over time. Since lower values indicated a better outcome, negative changes resulted in positive ranks and vice versa when using Wilcoxon Signed Rank Tests. Logistic regressions were performed to evaluate factors associated with lower CM at Q2 and change in CM from Q1 to Q2. All analyses were performed individually by gender with the critical α-value set at.05.

#### Procedures for logistic regression models

Four covariates - demographic, diseases, psychosocial, and activities - were entered in separate steps into cross-sectional and prospective logistic regression models using a stepwise forced entry method. The logistic regression models were performed at several steps. In the first step age, marital status, educational level, and subjective economy were entered. In the second step the musculoskeletal, cerebrovascular and eye condition indices were entered. In the third step subjective health, CES-D and social network were entered. These first three steps are included in Model 1 (Appendix S1). In the last step (Model 2 in Appendix S1), activities such as, sport activities, community activities and I-ADL outside home were entered.

Significant covariates obtained from the prospective and cross-sectional logistic regression models were then entered into prospective and cross-sectional models. This was done to potentially strengthen the fit of the models as measured by the pseudo-R^2^ values of Nagelkerke and Cox & Snell [20]. In these new models one covariate was introduced at each step starting with the one with the strongest p-value from Model 1 (Appendix S1) and continuing until the variable with the weakest significant p-value from Model 2 was entered (Appendix S1). This was done in the same way for both the prospective and the cross-sectional logistic regression models.

## Results

The mean age for the chosen sample in Q1 was 72.0 years (SD 2.62) for men and 72.7 years (SD 3.05) for women. In Q2 the mean age for men was 85.0 years (SD 2.62) and 85.6 years (SD 3.05) for women.

### Gender Differences and Prospective Changes with Respect to Covariates

Characteristics and prospective changes over time in study variables are presented in Table 1 separately by gender. Most changes over time were negative. However, men felt more part of a social network and women rated their subjective economy higher in Q2 compared with Q1.

**Table 1:** Characteristics of the sample at baseline in 1994 (Q1) and follow-up in 2007 (Q2).

		Q1^a^	Q2^b^	Prospective change Q1–Q2 z^d^	Gender difference Q1/Q2 χ^2^/z^e^
		Median/IQR^c^	Median/IQR^c^		
Age	Men	72/4	85/4	8.54***	1.48/1.29
	Women	72/4	85/4	9.75***	
Marital status 1–2	Men	1	1	3.36**	8.79**/17.97***
	Women	1	2	5.30***	
Educational level 1–6	Men	3/1	3/2	/	2.10*/2.36*
	Women	2/1	2/1	/	
Subjective economy 1–3	Men	1/1	1/1	0.92	1.72/0.39
	Women	1/1	1/1	−2.07*	
Cerebrovascular	Men	0/0	0/1	4.16***	−1.67/−1.87
conditions 0–4	Women	0/0	0/1	4.35***	
Eye conditions 0–3	Men	0/0	0/1	3.87***	1.29/3.95***
	Women	0/0	1/1	6.07***	
Musculo-skeletal	Men	0/1	0/1	0.19	−0.38/2.62**
conditions 0–6	Women	0/1	1/1	2.54*	
Subjective health 1–3	Men	1/1	2/1	3.43***	0.33/−0.26
	Women	1/1	2/1	3.33***	
CES-D 1–2	Men	1	1	0.47	3.92*/0.99
	Women	1	1	−0.90	
Social network 1–4	Men	3/1	3/1	−2.27*	−0.98/−0.22
	Women	3/1	3/1	−1.82	
Sport activities 1–2	Men	2	2	−1.71	0.23/0.89
	Women	2	2	−0.56	
Community	Men	12/4	13/4	2.35*	−0.91/−0.57
activities 1–15	Women	12/5	13/4.5	3.75***	
I-ADL outside home 1–5	Men	2/0	2/1	2.56*	−0.16/1.28
	Women	2/0	2/1	4.37***	

All covariates, except educational level, were coded so that lower values indicated a more positive outcome. Minus signs indicate a better outcome at Q2 with respect to prospective change and a better outcome for women with respect to gender differences.

Notes: *p<0.05, **p<0.01, ***p<0.001.

a Men = 100, women = 113.

b Men = 95, women = 125.

c Interquartile range (i.e., the difference between Q_3_–Q_1_) calculated for variables with more than two values.

d Analysed using Wilcoxon Signed Rank Test.

e Dichotomous variables analysed using Pearson’s χ^2^. All other variables analysed using Mann-Whitney U-test.

Compared to those individuals lost over the 13 years, the sample remaining in 2007 (Q2) had a significantly better situation in 1994 (Q1) than at the time of Q2 in regards to all study variables apart from subjective economy, depression and social network. Aside from being significantly younger, at the time of Q1 respondents were more often married, had a higher educational level, fewer health conditions, better subjective health and more often participated in activities outside home (*p*<.05). The majority (64%) were able to complete the questionnaire themselves at Q2. The remaining questionnaires were fully or partly completed by proxy due to poor vision (11%), musculoskeletal difficulties (7%) or for non-specified reasons (16%).

An additional internal loss was due to an error in the printing process (this loss was noticed after the first dissemination, but was corrected before reminders were distributed). The main question of this study regarding ability to transport beyond walking distance (Q2) was unfortunately among the lost pages. However, comparing those who had (n = 294) and those who had not (n = 63) answered this particular question, no significant differences were found with respect to the covariates from Q2 used in the present study.

### Community Mobility and Community Mobility Change

**Figure 2:**
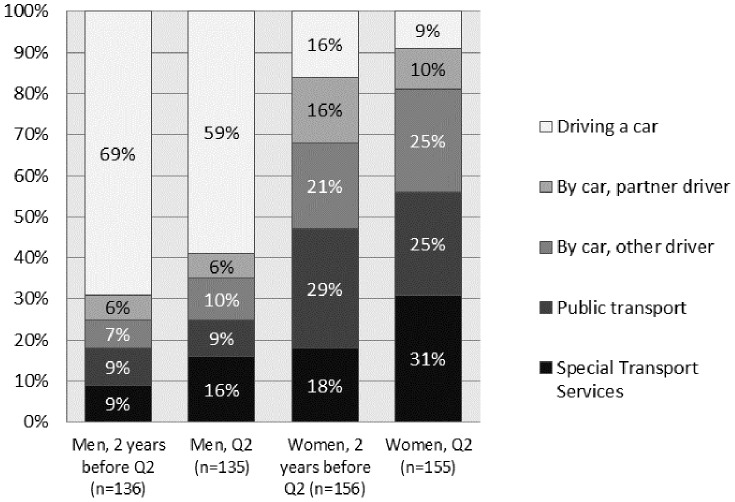
Distribution main mode of transport two years before follow-up and at follow-up (Q2).

Men decreased their car usage and increased their use of Special Transport Systems (STS; a demand-responsive mode of transportation provided to those eligible) over time (Figure 2). Similarly, women increased their use of STS over time, but decreased their use of private cars either as driver or passenger, as well as their use of PT. Men reported a more positive situation compared with women in both Q1 and Q2. As shown in Table 2, a larger share of men were drivers, compared to women, two years prior Q2 (*χ^2^* = 61.99, *p*<.001) and at the time of Q2 (χ^2^ = 64.52, *p*<.001). In contrast, a larger share of women used PT or STS two years before Q2 (χ^2^ = 19.00, *p*<.001) and at Q2 (χ^2^ = 20.72, *p*<.001).

**Table 2:** Gender differences with respect to driving a car compared to other modes of transport and use of public transport (PT) and Special transport system (STS) compared with other modes of transport.

	Men	Women
	2 years before Q2/at the time of Q2	2 years before Q2/at the time of Q2
Driving a car	n = 71 (60%)/n = 60 (50%)	n = 20 (14%)/n = 10 (7%)
Other modes of transport	n = 48 (40%)/n = 59 (50%)	n = 127 (86%)/n = 137 (93%)
PT/STS	n = 15 (13%)/n = 22 (19%)	n = 53 (36%)/n = 66 (45%)
Other modes of transport	n = 104 (87%)/n = 97 (81%)	n = 94(64%)/n = 81 (55%)

**Figure 3:**
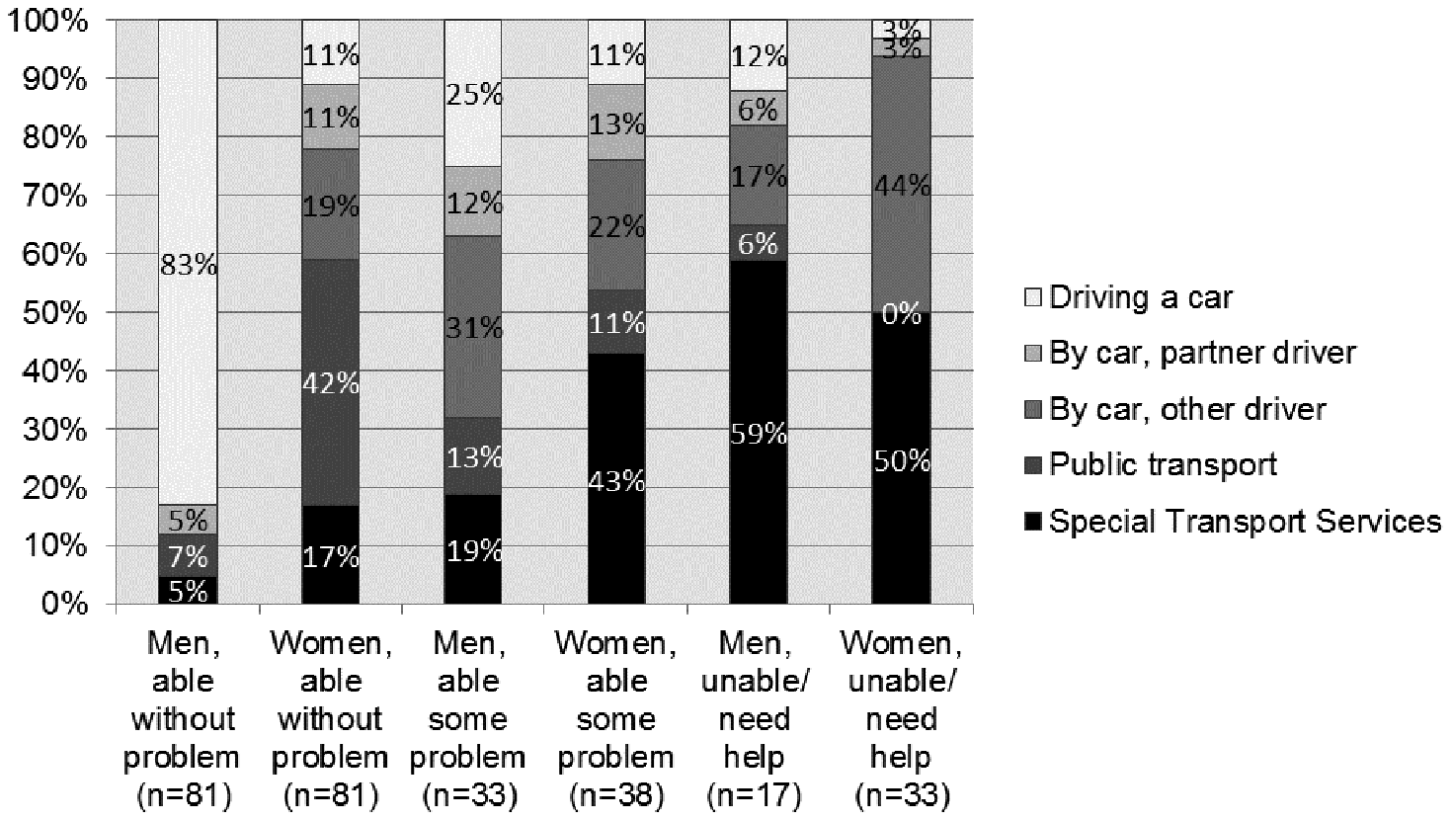
Ability to transport beyond walking distance relative to main mode of transport at follow-up (Q2).

Among car drivers, the majority, 79 (89%) men and 14 (93%) women, were below 88 years of age. Furthermore, the ability to transport oneself beyond walking distance was related to different modes of transportation for men and women, as shown in Figure 3.

Thirty-eight (40%) men and 54 (43%) women decreased their CM from Q1 to Q2, but 56 (47%) men and 65 (45%) women still reported independent mobility at Q2. Cross-sectionally (in Q2), CM was positively influenced by subjective health for both men and women, but also by few or non-existing depressive symptoms and more often taking part in sport activities for men, as well as more often taking part in I-ADL for women (Table 3). Prospectively, subjective health was an underlying factor for decreased CM over time among both men and women, together with higher age in women.

**Table 3:** Factors associated with CM in Q2 and decreased CM from Q1 to Q2.

		CM Q2^a^		Decreased CM from Q1 to Q2^b^	
		B^c^(SE)^d^	OR^e^(95% CI^f^)	B^c^(SE)^d^	OR^e^(95% CI^f^)
Men	Subjective health	−1.16 (0.61)	0.20** (0.06–0.66)	1.11 (0.47)	3.03* (1.21–7.56)
	Age	−0.19 (0.11)	0.83 (0.67–1.01)	0.15 (0.08)	1.16 (0.98–1.36)
	CES-D	−1.75 (0.74)	0.17* (0.04–0.73)	–	–
	Social network	−0.37 (0.40)	0.69 (0.31–1.53)	–	–
	I-ADL outside home	−0.23 (0.23)	0.80 (0.51–1.24)	0.54 (0.33)	0.10 (0.90−3.23)
	Sport activities	−1.31 (0.64)	0.27* (0.08–0.94)	–	–
Women	Subjective health	−0.97 (0.48)	0.38* (0.15–0.97)	1.01 (0.07)	2.73** (1.19–6.26)
	Age	−0.08 (0.08)	0.92 (0.79–1.07)	0.25 (0.07)	1.29* (1.11–1.49)
	CES-D	−0.18 (0.53)	0.84 (0.30–2.36)	–	–
	Social network	0.54 (0.29)	1.72 (0.98–3.02)	–	–
	I-ADL outside home	−0.71 (0.20)	0.49*** (0.34–0.73)	0.37 (0.33)	1.45 (0.76–2.76)
	Sport activities	−0.27 (0.53)	0.76 (0.27–2.16)		

Notes:

a Men (n = 95): Pseudo-R^2^ = 0.33 (Cox & Snell), 0.45 (Nagelkerke) Women (n = 125): Pseudo-R^2^ = 0.26 (Cox & Snell), 0.34 (Nagelkerke).

Predicts CM correctly for 80% of the men and 76% of the women.

b Men (n = 100): Pseudo-R^2^ = 0.12 (Cox & Snell), 0.17 (Nagelkerke) Women (n = 113): Pseudo-R^2^ = 0.19 (Cox & Snell), 0.25 (Nagelkerke).

Predicts CM change correctly for 68% of the men and 72% of the women.

c B =  regression coefficient, ^d^ SE =  standard error,^ e^ OR =  odds ratio, ^f^ CI = confidence interval.

^*^p<0.05, ^**^p<0.01, ^***^p<0.001.

## Discussion

The present study identified factors that were associated with CM and decreased CM over time among older men and women. Despite changes with respect to most of the variables in the present study, only subjective health predicted both cross-sectional and prospective CM, whereas self-reported common health conditions (e.g., stroke, heart failure, knee problems or cataract) were not associated with CM. This finding is intriguing, since CM is dependent on various body functions [2], [21], which in turn may be limited by different kinds of health conditions [22], [23]. However, a recent study found chronic health conditions to be associated with functional mobility limitations but not driving cessation [13]. This could be explained by the fact that driving may compensate for functional limitations by reducing the need to walk [24]. On the contrary, this is not the case when it comes to use of PT, which does not take the user all the way to the destination. Interestingly, PT did provide CM for several of the participants in the present study.

Despite increasing prevalence of physical health conditions [25], it is relevant to mention that activity limitations may remain unchanged and even decrease in older people [8], [26], [27] due to environmental improvements facilitating activity performance. It seems reasonable to assume that decreased activity limitations positively influence subjective health, and thereby offer one explanation to the findings.

Higher age was associated with decreased CM for women, which probably is explained by the fact that older women in general drive their own car to a lesser extent than older men [28], as shown in the present study. This is a cohort-effect affecting women negatively, since private transport by car has been found to compensate for declining functions, e.g., decreased walking ability and thereby potentially facilitating CM [24], [29]. As a consequence, special attention needs to be paid to older female non-drivers in order to facilitate driving by various modes of technical support, such as automatic rather than manual gear shifting [30]. This approach will likely benefit older men as well. When driving is no longer possible, PT becomes a vital CM option. Based on the findings of this study and others, PT-solutions need to be increasingly accessible and usable to support older peoples’ need [31]. To actively engage this user group in the design process of PT-systems may be an important step forward [32].

Over a two-year period in later-life, both men and women changed their main mode of transportation towards autonomous but not independent CM. For example, older men made transitions from driving a car to use of STS, while older women generally changed from car *and* PT to STS. These transitions may affect which trips are eventually being made and thereby which activities are possible to perform. In the present study, CM in women was associated with prioritising necessary trips like I-ADL outside the home. This finding reinforces the conclusions of a previous study [31], in which participants with reduced CM refrained from trips to friends and relatives, and potentially decreased their participation in health-promoting activities that provide subjective meaning and a sense of belonging [33]. Even if participation in I-ADL outside the home is important, such activities tend to also fulfill what people need to do rather, not merely what they want and find meaningful to do. However, for men, independent CM was associated with sports activities that probably also provide meaning and a sense of belonging. This finding further supports the notion that interventions aiming to promote or facilitate CM may need to differ, depending on target populations.

Removing the significant activity variable for both men (sport activities) and women (I-ADL outside home) in the logistic regression models did not alter the importance of the other self-reported activities. Hence, the identified gender differences were found to be consistent from this perspective. It should be mentioned that these activity differences between men and women may be cohort related, and decrease as future generations of men and women take more equal part in performing I-ADL. Nevertheless, it is important to acknowledge that reduced CM may decrease health-promoting participation in activities and that participation in activities outside home, as well as CM, ought to be addressed in health-promoting initiatives targeting older adults. Even if the present study was conducted based on the assumption that decreased CM may cause decreased participation in activities, the current study could not establish whether decreased participation in activities caused [6], [7] or merely was an effect [34] of decreased CM.

Another suggested gender difference was that lack of depression, relating also to subjective health [35], was cross-sectionally associated with independent CM in later-life for men only. Thus, interventions towards depressive symptoms may have the additional effect of promoting CM, at least for older men.

Based on our findings, interventions aiming to promote and facilitate CM must move beyond treatment of medical conditions, as well as interventions towards functional limitations, to instead target subjective health. This finding is promising, since health conditions in later life are more or less inevitable and probably difficult to successfully intervene against. However, subjective health could possibly be improved by interventions aiming to enable participation in activities that provide subjective purpose, meaning and belonging [33]. Moreover, individual and environmental compensation strategies have been found to influence and promote subjective health in relation to mobility [36], [37].

CM, like all other activities, is dependent on the person’s environmental context, in this case the community [38]. For example, being non-ambulant is strongly associated with environmental barriers [39]. The environmental improvements providing increased accessibility, like the Swedish transport system, may have influenced the findings of the present study, and are potentially fundamental to reducing the impact of health conditions on CM. Since the sample represented 51% of the 290 Swedish municipalities [40], and the survey included no questions relating to the environment, environmental assumptions must be made on a general level. Northern Europe and the Scandinavian countries generally have a system-oriented or integrated approach to accessible built environments and PT [41]. As our findings are context dependent they are therefore only generalizable to similar contexts.

No objective health measures were used in the present study. Instead, health conditions were self-reported. However, some of these were defined as problems (such as hip problems and knee problems) rather than actual diagnoses, and therefore probably easier to self-report accurately. In fact, self-reports provide more information than medical records on conditions which predominantly include subjective symptoms [42].

Furthermore, the dependent variable in the present study, the ability to transport beyond walking distance, was defined by the participants themselves and not based on specific distances. However, data on distances from home to CM relevant activities were not recorded in the present study. Nevertheless, it is reasonable to assume that the distance covered may have decreased over the 13 years between Q1 and Q2. To use a valid test with a specific walking range [43] may have provided more exact data in regards to distance. However, self-reported ability to transport beyond walking includes more than the walking ability per se, but also the ability and possibility to use private and PT. Thus, it seems reasonable to assume that a question capturing participants’ performance in their current environment with respect to CM at both points in time would capture the relevant information. Some may consider the use of self-reported performance rather than professional assessments a limitation, however, self-reported and externally assessed mobility have been shown to be highly associated in a recent study [44]. Another limitation of the present study is that no objective economic measurement was used. However, previous studies have found subjective socio-economic status to be more strongly associated with health-related factors than any objective measures [45].

Twin samples have the advantage of being gender balanced. To avoid twin bias analyses were conducted separately by gender. Gender differences identified in a sample of unlike-sex twins are also most likely to also exist in a general population. With respect to health status and functioning, previous studies have shown that twins are comparable to non-twins in later life [46]. Thus, the current results are assumed to be applicable to the general population. Moreover, the well-known dilemma in gerontology research, i.e., that the healthiest have survived, applies also to the present study. Additional studies including a larger sample of older people and in other environmental contexts are needed to further explore the association between CM and subjective health.

### Conclusions

Decreased and independent CM was associated with subjective health for both men and women rather than by self-reported health conditions. All other significant factors differed by gender. Societal measures and individual interventions aiming to improve CM or prevent CM reductions among older adults must look beyond objective measures of symptoms and functional limitations, and acknowledge older persons’ subjective perspectives of health.

## Supporting Information

Appendix S1 **Factors associated with CM at Q2 and decreased CM from Q1 to Q2.**
(DOCX)
