# Supplementary material for: Changes in Community Mobility in Older Men and Women. A 13-Year Prospective Study
Source: PLoS One. 2014 Feb 7;9(2):e87827. doi: 10.1371/journal.pone.0087827 (PMC3917836; doi:10.1371/journal.pone.0087827)
Supplement: Appendix S1 — Factors associated with CM at Q2 and decreased CM from Q1 to Q2. (DOCX) [file pone.0087827.s001.docx]

Appendix S1, Factors associated with CM at Q2 and decreased CM from Q1 to Q2.

|  | |  | CM Q2 – Model 1^a^ | | CM Q2 – Model 2^b^ | | Decreased CM from Q1 to Q2 - Model 1^c^ | | Decreased CM from Q1 to Q2 - Model 2^d^ | |
| --- | --- | --- | --- | --- | --- | --- | --- | --- | --- | --- |
|  | |  | B^e^(SE)^f^ | OR^g^(95% CI^h^) | B^e^(SE)^f^ | OR^g^(95% CI^h^) | B^e^(SE)^f^ | OR^g^(95% CI^h^) | B^e^(SE)^f^ | OR^g^(95% CI^h^) |
| Men | Age | | -0.23 (0.11) | 0.79* (0.64-0.99) | -0.21 (0.12) | 0.81 (0.64-1.03) | 0.14 (0.09) | 1.15 (0.97-1.37) | 0.16 (0.09) | 1.18 (0.98-1.41) |
|  | Marital status | | -0.27 (0.53) | 0.76 (0.27-2.13) | -0.37 (0.52) | 0.69 (0.23-2.09) | 0.09 (0.60) | 1.09 (0.34-3.53) | -0.10 (0.64) | 0.90 (0.26-3.17) |
|  | Educational level | | 0.12 (0.20) | 1.13 (0.77-1.66) | 0.11 (0.20) | 1.12 (0.75-1.66) | 0.02 (0.18) | 1.02 (0.72-1.45) | 0.20 (0.20) | 1.22 (0.83-1.80) |
|  | Subjective economy | | 0.28 (0.44) | 1.32 (0.55-3.16) | 0.28 (0.47) | 1.33 (0.53-3.30) | 0.77 (0.43) | 2.16 (0.94-5.00) | 0.85 (0.46) | 2.34 (0.96-5.73) |
|  | Musculoskeletal conditions | | 0.22 (0.35) | 1.24 (0.63-2.46) | 0.27 (0.37) | 1.31 (0.64-2.69) | 0.08 (0.32) | 1.09 (0.58-2.04) | -0.02 (0.35) | 0.97 (0.49-1.93) |
|  | Cerebrovascular conditions | | -0.18 (0.27) | 0.83 (0.49-1.40) | -0.21(0.28) | 0.81 (0.47-1.41) | 0.25 (0.44) | 1.29 (0.55-3.02) | 0.31 (0.44) | 1.36 (0.57-3.26) |
|  | Eye conditions | | 0.10 (0.37) | 1.10 (0.53-2.29) | 0.20 (0.39) | 1.23 (0.57-2.64) | 0.14 (0.55) | 1.15 (0.39-3-38) | 0.34 (0.56) | 1.41 (0.47-4.24) |
|  | Subjective health | | -1.99 (0.62) | 0.14*** (0.04-0.46) | -1.62 (0.67) | 0.200.05-0.74) | 1.45 (0.52) | 4.25** (1.54-11.70) | 1.34 (0.55) | 3.83* (1.30-11.28) |
|  | CES-D | | -1.50 (0.70) | 0.22* (0.06-0.88) | -1.75 (0.81) | 0.17* (0.04-0.85) | -0.43 (0.65) | 0.65 (0.18-2.32) | -0.44 (0.68) | 0.64 (0.17-2.46) |
|  | Social network | | -0.24 (0.42) | 0.79 (0.35-1.78) | -0.41 (0.48) | 0.66 (0.26-1.69) | 0.63 (0.43) | 1.88 (0.81-4.36) | 0.84 (0.48) | 2.32 (0.91-5.95) |
|  | Sport activities | | - | - | -1.39 (0.68) | 0.25* (0.07-0.95) | - | - | 0.82 (0.64) | 2.26 (0.64-7.96) |
|  | Community activities | | - | - | 0.02 (0.14) | 1.02 (0.77-1.35) | - | - | 0.02 (0.10) | 1.02 (0.84-1.25) |
|  | I-ADL outside home | | - | - | -0.24 (0.24) | 0.78 (0.49-1.25) | - | - | 0.78 (0.36) | 2.18* 1.07-4.43) |
|  |  | |  |  |  |  |  |  |  |  |
| Women | Age | | -0.16 (0.07) | 0.85* (0.74-0.98) | -0.09 (0.08) | 0.91 (0.77-1.07) | 0.24 (0.09) | 1.28** (1.08-1.51) | 0.24 (0.09) | 1.27** ^(^1.07-1.52) |
|  | Marital status | | -0.13 (0.47) | 0.88 (0.35-2.22) | -0.36 (0.54) | 0.70 (0.24-1.99) | 0.73 (0.48) | 2.07 (O,81-5.33) | 0.82 (0.51) | 0.73 (0.46-1.17) |
|  | Educational level | | 0.25 (0.20) | 1.28 (0.86-1.91) | 0.09 (0.22) | 1.10 (0.72-1.68) | -0.34 (0.23) | 0.71 (0.46-1.11) | -0.31 (0.24) | 0.99 (0.48-2.03) |
|  | Subjective economy | | -0.06 (0.35) | 0.94 (0.48-1.87) | -0.02 (0.37) | 0.98 (0.47-2.04) | 0.06 (0.35) | 1.06 (0.54-2.10) | -0.01 (0.37) | 2.28 (0.83-6.23 |
|  | Musculo-skeletal conditions | | -0.30 (0.23) | 0.74 (0.47-1.16) | -0.39 (0.25) | 0.68 (0.41-1.12) | -0.08 (0.31) | 0.92 (0.51-1.68) | 0.02 (0.32) | 1.02 (0.55-1.90) |
|  | Cerebrovascular conditions | | -0.15 (0.29) | 0.86 (0.49-1.51) | 0.00 (0.33) | 0.82 (0.46-1.43) | -0.80 (0.63) | 0.45 (0.13-1.55) | -0.67 (0.67) | 0.51 (0.14-1.90) |
|  | Eye conditions | | 0.08 (0.29) | 1.08 (0.62-1.90) | 0.17 (0.31) | 1.18 (0.64-2.18) | 0.12 (0.47) | 1.13 (0.45-2.85) | 0.03 (0.49) | 1.03 (0.40-2.68) |
|  | Subjective health | | -0.66 (0.47) | 0.52 (0.21-1.28) | -0.95 (0.54) | 0.39 (0.14-1.10) | 1.00 (0.48) | 2.72* (1.06-7.02) | 0.87 (0.50) | 2.38 (0.89-6.37) |
|  | CES-D | | -0.52 (0.49) | 0.59 (0.23-1.57) | -0.04(0.57) | 0.96 (0.31-2.95) | 0.18 (0.51) | 1.20 (0.44-3.23) | 0.19 (0.53) | 1.21 (0.43-3.41) |
|  | Social network | | 0.55 (0.28) | 1.17* (1.01-2.99) | 0.63 (0.32) | 1.88* (1.01-3.50) | -0.23 (0.37) | 0.79 (0.39-1.63) | -0.22 (0.38) | 0.80 (0.38-1.69) |
|  | Sport activities | | - | - | -0.36 (0.57) | 0.70 (0.23-2.13) | - | - | 0.26 (0.59) | 1.29 (0.41-4.10) |
|  | Community activities | | - | - | 0.07 (0.09) | 1.07 (0.89-1.28) | - | - | 0.15 (0.08) | 1.17 (0.99-1.38) |
|  | I-ADL outside home | | - | - | -0.76 (0.21) | 0.47*** (0.31-0.71) | - | - | 0.52 (0.36) | 1.68 (0.83-3.40) |

Notes: ^a^ Men (n=95): Pseudo-R^2^= 0.29 (Cox & Snell), 0.40 (Nagelkerke). Women (n=125): Pseudo-R^2^= 0.18 (Cox & Snell), 0.24 (Nagelkerke). Predicts CM correctly for 77% of the men and 63% of the women.

^b^ Men (n=95): Pseudo-R^2^= 0.35 (Cox & Snell), 0.47 (Nagelkerke). Women (n=125): Pseudo-R^2^= 0.28 (Cox & Snell), 0.38 (Nagelkerke). Predicts CM correctly for 80% of the men and 74% of the women.

^c^ Men (n=100): Pseudo-R^2^= 0.15 (Cox & Snell), 0.20 (Nagelkerke). Women (n=113): Pseudo-R^2^= 0.24 (Cox & Snell), 0.32 (Nagelkerke). Predicts CM change correctly for 68% of the men and 71% of the women.

^d^ Men (n=100): Pseudo-R^2^= 0.20 (Cox & Snell), 0.27 (Nagelkerke). Women (n=113): Pseudo-R^2^= 0.27 (Cox & Snell), 0.37 (Nagelkerke). Predicts CM change correctly for 71% of the men and 76% of the women.

^e^ B= regression coefficient, ^f^SE= standard error, ^g^OR= odds ratio, ^h^CI=confidence interval.

^*^p<0.05, ^**^p<0.01, ^***^p<0.001.
